# HMGB2 is associated with malignancy and regulates Warburg effect by targeting LDHB and FBP1 in breast cancer

**DOI:** 10.1186/s12964-018-0219-0

**Published:** 2018-02-20

**Authors:** Deyuan Fu, Jing Li, Jinli Wei, Zhengquan Zhang, Yulin Luo, Haosheng Tan, Chuanli Ren

**Affiliations:** 1 Department of Thyroid and Breast Surgery, Northern Jiangsu People’s Hospital and Clinical Medical College of Yangzhou University, No.98 Nantong West Road, Yangzhou, 225001 China; 2 Departments of CyberKnife, Huashan Hospital, Fudan University, No.525,Hongfeng Road, Shanghai, 200032 China; 3 The Clinical Medical Testing Laboratory, Northern Jiangsu People’s Hospital and Clinical Medical College of Yangzhou University, Yangzhou, 225001 China; 4 Departments of CyberKnife, Huashan Hospital, Fudan University, No.525,Hongfeng Road, Shanghai, 201206 China

**Keywords:** HMGB2, Breast cancer, Proliferation, Warburg effect

## Abstract

**Background:**

High-mobility group box 2 (HMGB2) is implicated in tumorigenesis in various cancers. However, the clinical significance of HMGB2 signaling in human breast cancer progression remains unknown.

**Methods:**

We investigated HMGB2 expression in 185 cases of primary breast cancer and matched normal breast tissue specimens, and explored the underlying mechanisms of altered HMGB2 expression as well as the impact of this altered expression on breast cancer growth and on aerobic glycolysis using in vitro and animal models of breast cancer.

**Results:**

HMGB2 was more highly expressed in tumor-cell nuclei of breast cancer cells than in the adjacent normal breast tissues (*P* < 0.05). Higher HMGB2 expression correlated with larger tumor size (*P* = 0.003) and advanced tumor stage (*P* = 0.033). A Cox proportional hazards model revealed that HMGB2 expression was an independent prognostic factor for breast cancer after radical resection (*P* < 0.05). Experimentally, knockdown of HMGB2 expression by stable transfected shRNA significantly decreased the growth and glycolysis of breast cancer cells both in vitro and in mouse models. Mechanically, promotion of breast cancer progression by HMGB2 directly and significantly correlated with activation of LDHB expression and inactivation of FBP1 expression.

**Conclusions:**

These results disclose a novel role for HMGB2 in reprogramming the metabolic process in breast cancer cells by targeting LDHB and FBP1 and provide potential prognostic predictors for breast cancer patients.

## Background

Breast cancer is the most common type of cancer among women worldwide, accounting for 29.63% of new cancer cases in 2017 [1]. It is estimated that about 40,610 women will die from breast cancer in 2017 in the USA [1]. Despite advances in prevention, diagnosis, and treatment of breast cancer, the recurrence and mortality rate remain high [2]. Considering the heterogeneity of breast cancer, numerous efforts have been made to explore the underlying biological and pathological characteristics [3]. The molecular mechanisms of carcinogenesis are complex due to variable combinations of aberrant protein expression, gene changes, and miRNA deregulation. Therefore, numerous studies have focused on screening for novel diagnostic and prognostic biomarkers and therapeutic targets in breast cancer [4–7].

High-mobility group box (HMGB) proteins are ubiquitous, the second most abundant proteins in humans, and exert global genomic functions in establishing active or inactive chromatin domains [8]. HMGB1 and HMGB2 are highly conserved (with > 80% amino acid identity) and have indistinguishable biological properties, including binding to DNA without sequence specificity [9–11]. HMGB1 has been reported to be involved in diseases such as arthritis, sepsis, and cancer [12]. HMGB1 overexpression has been reported in a variety of human cancers, including hepatocellular carcinoma [9], pancreatic cancer [8], leukemia [13], and breast cancer [14, 15]. Despite extensive characterization of the diverse roles of HMGB1 in cancer, much less is known about the signaling pathways of HMGB2, especially its relevance in carcinogenesis [9]. Recently, it has been reported that HMGB2-mediated cell proliferation and radiosensitivity via retinoblastoma-interaction-dependent and -independent mechanisms [16]. HMGB2 also induced tamoxifen resistance via interactions with the ER complex at specific target genes [17]. However, little is known about HMGB2 expression and its prognostic significance in breast cancer.

The present study investigated the clinicopathologic significance of HMGB2 expression in a large number of breast cancer cases via an immunohistochemistry study. Furthermore, we analyzed in vitro the effects of HMGB2 knockdown on cell growth and on the Warburg effect, which is thought to be fundamental to tumor growth and progress. Our study demonstrates that high HMGB2 predicts a poor prognosis by promoting cell proliferation and glycolysis in breast cancer cells.

## Methods

### Patients’ samples

Breast cancer specimens from patients who underwent intentionally curative surgical resection from January 2005 to December 2016 were obtained to validate the clinical significance of HMGB2 protein expression. Tumor tissues were histopathologically verified as adenocarcinoma, and noncancerous tissues were confirmed as negative.

Patients’ demographic and clinicopathological variables, including age, sex, primary site, histological type, Tumor-node-metastases(TNM) stage, pathological grade, ER/PR/Her-2 status, and treatment type, were recorded prospectively. All patients were restaged according to the 8th edition of the TNM-UICC/AJCC classification. Patients were revisited regularly according to The National Comprehensive Cancer Network (NCCN) guidelines. As this study describes the prognosis of patients with breast cancer, analyses of overall (OS) and disease free survival (DFS) were ascertained. The OS was defined as the time from treatment to death from any cause, and the DFS was defined as the time from treatment to the first recurrence or death. Patients who were alive at last follow-up were censored for analysis.

### Immunohistochemical staining (IHC)

IHC staining was performed according to standard methods [18, 19]. HMGB2 anti-human rabbit antibody was used at a dilution of 1:100 (14597–1-APl, Proteintech); PBS was used as a negative control. The degree of immunostaining of the sections was scored separately by two pathologists who were blind to the clinical and histopathologic features. The scores were combined to obtain the proportion of positively stained tumor cells and the staining intensity. The number of positive tumor cells was graded as follows: ‘-’ (< 5% positive tumor cells), ‘+’ (5–25% positive tumor cells), ‘++’ (26–50% positive tumor cells), and ‘+++’ (> 50% positive tumor cells). The staining intensity was recorded as follows: ‘-’ (no staining), ‘+’ (weak staining, light yellow), ‘++’ (moderate staining, yellowish brown), and ‘+++’ (strong staining, brown). For further data analysis, the samples scored ‘–’ or ‘+’ were considered to present a low level of HMGB2 expression, while the samples scored ‘++’ or ‘+++’ were considered to present a high level of HMGB2 expression [18, 20].

### Cell culture and transfection

The human breast cancer cell lines (MDA-MB-231 and MCF-7) were obtained from the American Type Culture Collection (ATCC; Rockville, MD, USA) and kept in RPMI-1640 medium with 10% fetal bovine serum (FBS) (Gibco, Carlsbad, CA, USA), 1% 100 U/ml penicillin, and 1% 100 mg/ml streptomycin sulfates. The cells were incubated in humidified incubators with 5% CO2 at 37̊C.

### Lentivirus production and stable cell line selection

To generate shRNA expression constructs against HMGB2, a pLKO.1 TRC cloning vector (Plasmid10878, Addgene, Cambridge, MA, USA) was employed. The 21 bp shRNA target against HMGB2 was 5′- GCTCAATACTAGCTTCAGTAT -3′. pLKO.1-scramble shRNA (Plasmid1864, Addgene) with limited homology, with any known sequences in humans used as a negative control. Lentiviral particles were produced by co-transfection of pLKO.1-shHMGB2 constructs with psPAX2 and pMD2.G into HEK-293 T cells in a ratio of 4:3:1. Cell lines were obtained by infection of MDA-MB-231 and MCF-7 cells with lentiviral particles followed by puromycin selection.

### Quantitative real-time PCR

Total RNA was extracted from tissues and cells using a TRIzol reagent (Invitrogen), and then miRNAs were reverse-transcribed to cDNA using a reverse transcription kit (RR036A, Takara, Tokyo, Japan). Quantitative real-time PCR (qRT-PCR) was performed using a SYBR-Green PCR kit on an ABI 7900 Fast Real-Time PCR system. The expression of the target gene mRNA was normalized to β-actin. All experiments were carried out in triplicate. The RQ value was used to calculate the relative expression of the genes.

### Western blotting

Cells were extracted with RIPA lysis buffer (Biyuntian, Hangzhou, China). Protein lysates were then separated by 10% sodium dodecyl sulfate-poly acrylamide gel electrophoresis (SDS-PAGE) and transferred to polyvinylidene fluoride (PVDF) membranes (Millipore, Billerica, MA, USA). After blocking with 5% non-fat milk for 2 h at room temperature, the membranes were incubated with the primary antibodies HMGB2 (14597–1-APl, Proteintech) and β-actin (Abcam, ab133626, 1:5000) overnight at 4 °C. The membranes were then incubated in HRP-linked secondary antibodies (anti-rabbit IgG; Cell Signaling Technology, Danvers, MA, USA; 1:7500) for 2 h. Western blotting signals were detected using the ECL Plus kit (Biyuntian). Each experiment was repeated 3 times independently.

### Cell proliferation assay and clonogenic assay

The proliferation ability of breast cancer cells was assessed using an established CCK8 assay [18]. To determine their clonogenic ability, cells were transplanted to a 6-well culture dish at a density of 200 cells per well and allowed to grow for 10–14 days to form colonies. Cells were stained with 0.1% crystal violet after being fixed with methanol. All visible colonies were counted manually. Values were normalized using the control value.

### Measurement of glucose assumption

The Glucose Uptake Colorimetric Assay Kit (Biovision, Milpitas, CA, USA) was used to examine the glycolysis process in breast cancer cells. Briefly, cells were washed twice by phosphate buffered saline (PBS) and then incubated in Krebs buffer without glucose for 30 min. The Krebs buffer was then replaced with a Krebs buffer containing 10 mM glucose spiked with 10 mCi of 5-^3^H-glucose. In 1 h, the ^3^H2O generated was separated chromatographically and measured using a liquid scintillation counter.

### Measurement of lactate production

Cells were plated in a 96-well plate and grown to approximate 80% confluence. The old medium was discarded, and the cells were washed twice and moved to fresh medium. The cells were incubated for one more hour, and the medium was collected. Lactate in the medium was measured with fluorescence-based assay kits (BioVision). Production of lactate was estimated by calculating the difference between lactate concentrations in the medium before and after culturing. Cell number was counted using a Coulter particle analyser [21].

### Animal experiments

All procedures involving mice were conducted in accordance with YangZhou University Animal Care guidelines. All efforts were made to minimize animal suffering, to reduce the number of animals used, and to utilize possible alternatives to in vivo techniques. Tumor cells (5 × 10^6^ per mouse for MCF-7-shHMGB2 and scramble) were injected in the forelimbs of each 4–6 week old Balb/C athymic nude mouse. Animals were euthanized 6 weeks later. Tumor size was measured by a slide caliper and tumor volume was determined by the formula 0.44 × A × B^2^ (A indicates tumor base diameter one direction and B the corresponding perpendicular value). Tissue was fixed in 10% buffered formalin, immersed in an ascending series of alcohols, and paraffin embedded. 4 μm sections were cut and stained with hematoxylin and eosin (H & E).

### Statistical analysis

Two-tailed χ2 test was used to evaluate the expression difference between the clinicopathological features and HMGB2 expression. Survival curves were estimated by Kaplan-Meier analysis, and *P* values were calculated by the log rank test. Univariate Cox proportional hazards regressions were applied to estimate the individual hazard ratio (HR) for the DFS and OS. The HR with 95% confidence interval (CI) was measured to estimate the hazard risk of each individual factor. All experiments were performed independently with three replicates. All *P* values were two-sided, and *P* < 0.05 was considered statistically significant. All statistical calculations were performed using SPSS 17.0.

## Results

### Patient characteristics

A total of 185 eligible female patients with breast cancer were included in IHC staining study (Table 1). The median age was 56, ranging from 29 to 80 years old. There were 26 (14.05%), 75 (40.54%), and 84 (45.41%) patients diagnosed with stage I, II, and III, respectively. The median follow-up time was 62, ranging from 12 to 89 months. At last follow up, 68 (36.8%) patients had been diagnosed with tumor relapse, and 53 (28.6%) patients had died.

**Table 1:** Comparison of baseline clinical characteristics based on HMGB2 expression level

			HMGB2		*χ2* value	*P* value
Variable		n	Low (%)	High (%)		
Age					0.140	0.904
	≤55	86	34(45.9)	52(46.8)		
	> 55	99	40(54.1)	59(53.2)		
Tumor Diameter					9.05	0.003
	< 2 cm	73	39(52.7)	34(30.6)		
	≥2 cm	112	35(47.3)	77(69.4)		
Grade					2.016	0.365
	I	96	43(58.1)	53 (47.7)		
	II	70	25(33.8)	45(40.5)		
	III	19	6(8.1)	13(11.7)		
Stage					6.801	0.033
	I	26	12(16.2)	14(12.6)		
	II	75	37(50.0)	38(34.2)		
	III	84	25(9.8)	59(53.2)		
Subtypes					4.733	0.129
	Luminal A	88	40(54.1)	48(43.2)		
	Luminal B	42	13(17.6)	29(26.1)		
	Basal-like	33	10(13.5)	23(20.7)		
	Her-2+	22	11(14.9)	11(9.9)		
ER Status					1.20	0.273
	Negative	48	16(21.6)	32(28.8)		
	Positive	137	58(78.4)	79(71.2)		
PR Status					2.183	0.140
	Negative	72	24(32.7)	48(43.2)		
	Positive	113	50(67.6)	63(56.8)		
HER-2/neu Status					0.498	0.480
	Negative	152	59(79.7)	93(83.8)		
	Positive	33	15(20.3)	18(16.2)		

### High HMGB2 expression is correlated with breast cancer progression

**Fig. 1:**
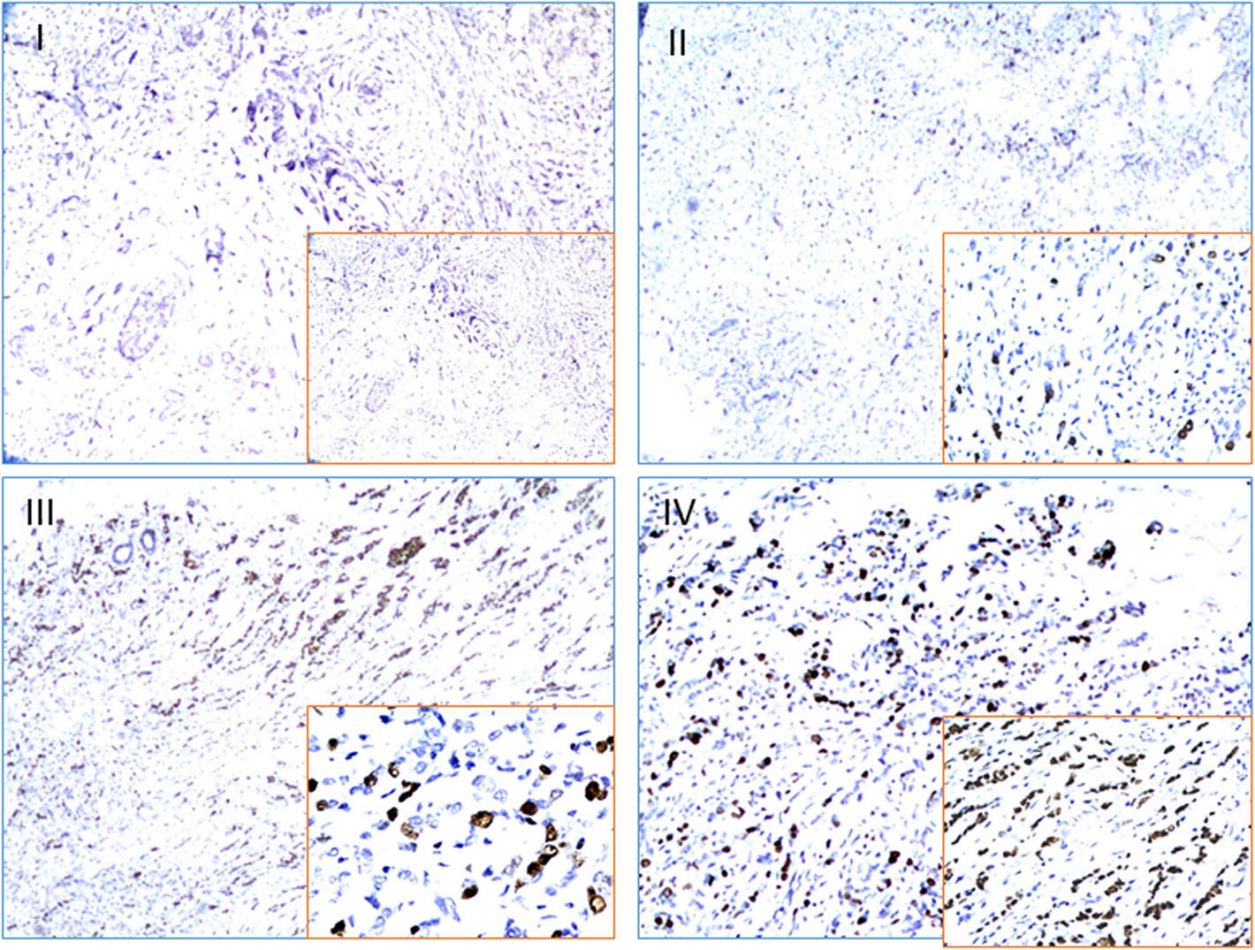
Representative samples with HMGB2 staining (primarily in nuclei). I-IV represent HMGB2 negative (I), weak (II), moderate (III) and strong (IV) staining in breast cancer tissues (Original magnification: 200× for the inserts, 40× for the whole)

HMGB2 expression was identified in the nucleus of breast cancer cells. Representative immunohistochemical images demonstrating negative, weak, moderate and high HMGB2 expression in breast cancer tissues are shown in Fig. 1. HMGB2 was expressed much more highly in breast cancer (111/185, 60.00%) than in noncancerous breast tissues (36/185, 19.46%) (*P* < 0.01). The percentage of high HMGB2 expression was observed in 12.6% of stage I patients, 34.2% of stage II patients, and 53.2% of stage III patients, and these differences were statistically significant (*P* = 0.033; Table 1). The HMGB2 expression was significantly higher in patients with tumors ≥2 cm diameter than those < 2 cm (*P* = 0.003). The result indicates that HMGB2 may affect the initiation, progression, and metastasis of breast cancer.

### Higher HMGB2 expression is correlated with poor survival in breast cancer patients

**Fig. 2:**
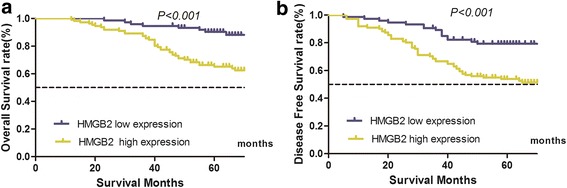
IHC staining demonstrates high HMGB2 expression correlates with lower survival in patients with breast cancer. **a** 5-year overall survival (OS) in the high and low HMGB2 expression groups wass 65.2% and 90.3%,respectively (χ2 = 17.75, *P* < 0.001). **b** 5-year disease-free survival (DFS) in the high and low HMGB2 expression groups was 53.9% and 79.3%, respectively (χ2 = 13.571, *P* < 0.001)

We evaluated the correlations between expression of nuclear HMGB2 protein and patients’ 5-year OS or DFS. Our data revealed a lower OS in patients with higher HMGB2 expression (high vs. low expression: 65.2% vs. 90.3%, χ^2^ = 17.75, *P* < 0.001; Fig. 2a). Similarly, a higher DFS correlated with higher HMGB2 expression (high vs. low expression: 53.9% vs. 79.3%, χ^2^ = 13.571, *P* < 0.001; Fig. 2b). Furthermore, multivariate Cox regression analysis showed that HMGB2 expression is an independent factor predicting both patients’ OS (HR: 3.009, 95% CI: 1.393–6.500, *P* = 0.005; Table 2) and DFS (HR: 1.985, 95% CI: 1.094–3.601, *P* = 0.024; Table 3). Our data implies that HMGB2 may serve as a potential prognostic marker in breast cancer clinical practice.

**Table 2:** Univariate and multivariate survival analyses of HMGB2 expression and overall survival for patients with breast cancer

Variable	Univariate analysis		Multivariate analysis	
	HR (95%CI)	*P*	HR (95%CI)	*P*
Age	1.022 (0.997–1.048)	0.090		NI
Tumor Diameter	3.748(1.829–7.681)	< 0.001	2.493(1.194–5.203)	0.015
Grade	1.955(1.346–2.840)	< 0.001	1.654(1.115–2.452)	0.012
Stage	2.991(1.790–4.999)	< 0.001	1.843(1.476–2.738)	0.026
Subtypes	1.206(0.952–1.528)	0.121		NI
ER Status	0.532(0.305–0.927)	0.026	0.814(0.459–1.444)	0.482
PR Status	1.003(0.578–1.740)	0.992		NI
HER-2/neu Status	1.055(0.530–2.102)	0.879		NI
HMGB2	4.372(2.059–9.280)	< 0.001	3.009 (1.393–6.500)	0.005

NI: not included in multivariate survival analysis

**Table 3:** Univariate and multivariate survival analyses of HMGB2 expression and disease free survival for patients with breast cancer

Variable	Univariate analysis		Multivariate analysis	
	HR (95%CI)	*P*	HR (95%CI)	*P*
Age	1.027 (1.004–1.050)	0.020	1.028(1.005–1.051)	0.016
Tumor Diameter	2.722(1.534–4.832)	0.001	2.040(1.132–3.675)	0.018
Grade	1.899(1.359–2.654)	< 0.001	1.520(1.069–2.162)	0.020
Stage	2.705(1.762–4.152)	< 0.001	2.115(1.385–3.231)	0.001
Subtypes	1.279(1.014–1.573)	0.019	1.038(0.820–1.313)	0.758
ER Status	0.595(0.360–0.985)	0.043	0.763(0.436–1.334)	0.343
PR Status	0.856(0.530–1.385)	0.527		NI
HER-2/neu Status	0.808(0.413–1.580)	0.533		NI
HMGB2	2.719(1.573–4.953)	< 0.001	1.985 (1.094–3.601)	0.024

NI: not included in multivariate survival analysis

### Silencing HMGB2 expression significantly reduced proliferation and colony formation

**Fig. 3:**
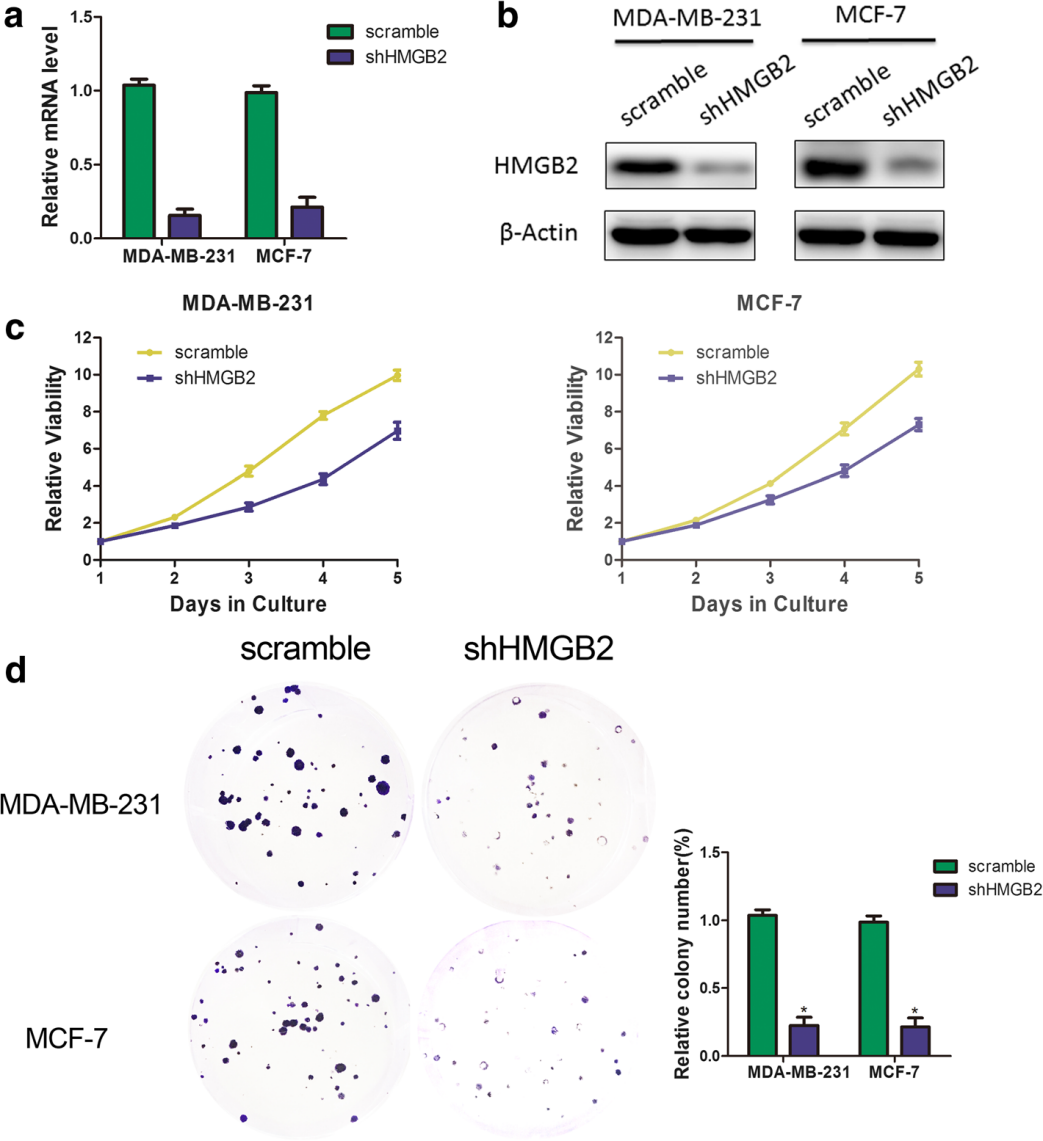
HMGB2 was associated with viability of breast cancer cells in vitro. Efficiency of HMGB2 knockdown in breast cancer cell lines was measured by RT-PCR (**a**) and western blot (**b**). Influence of HMGB2 expression on viability of breast cancer cells was measured by CCK-8 assay (**c**) and clone formation (**d**). Values are presented as mean ± standard deviation of 3 independent experiments.*P < 0.05

To assess the role of HMGB2 in regulating breast cell proliferation, HMGB2 expression was silenced by lentivirus mediated transfection. Two shRNA oligos could effectively decrease HMGB2 expression at both the transcription level and the protein level in MDA-MB-231 and MCF-7 cells (Fig. 3a and b). To assess the role of HMGB2 in cell viability, a CCK-8 proliferation assay was performed, and our results demonstrated that inhibition of HMGB2 expression attenuated cell proliferation (Fig. 3c). Furthermore, colony formation assay demonstrated that silencing HMGB2 expression resulted in the reduction of clone formation capacity in MDA-MB-231 and MCF-7 cells (Fig. 3d). Taken together, these results validated HMGB2 as a positive regulator of cell proliferation in breast cancer tissues.

### Effects of HMGB2 on aerobic glycolysis in breast cancer cells in vitro

**Fig. 4:**
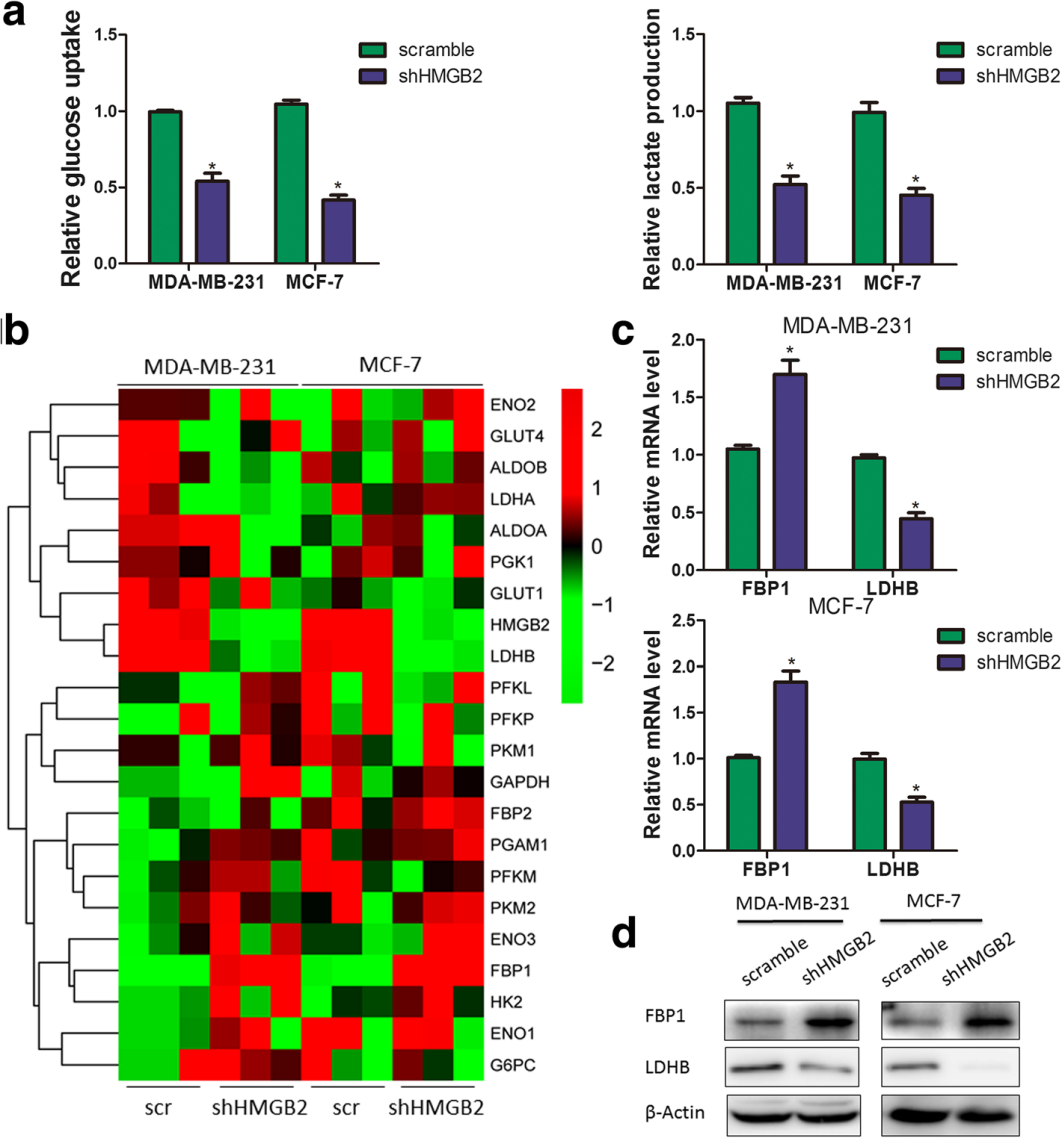
Effects of HMGB2 on aerobic glycolysis in breast cancer cells in vitro. **a** Silencing HMGB2 expression in MDA-MB-231 and MCF-7 cells significantly decreased glucose utilization and lactate production. Glycolysis is a multi-step enzymatic reaction involving with a series of rate-limiting enzymes, (**b**) Heatmap of differentially expressed glycolytic enzymes genes in HMGB2 knockdown breast cancer cells using qRT-PCR with results normalized by Z-score. Each column represents a specimen, denoted above, and each role represents a gene, which is denoted on the right. Red color indicates genes that were upregulated and green color indicates genes that were downregulated. Results were further validated by RT-PCR (**c**) and Western blot (**d**) in HMGB2 stable knockdown cells

Tumor formation and progression requires transformation of the glucose metabolism in order to fuel its malignant growth [19, 22–24]. To determine the effect of altered HMGB2 expression on aerobic glycolysis in breast cancer cells, we calculated the glucose utilization and lactate concentrations of the HMGB2 stable knockdown cells. Silencing HMGB2 expression strongly decreased glucose utilization and lactate production in MDA-MB-231 and MCF-7 cells (Fig. 4a). Glycolysis is a multi-step enzymatic reaction involving a series of rate limiting enzymes [19, 25]. To assess the effect of HMGB2 on the expression of the rate limiting enzymes involved in glycolysis, we carried out quantitative real time PCR to examine the transcription levels of these enzymes. Silencing HMGB2 expression significantly decreased the LDHB mRNA level, while upregulating the FBP1 mRNA level (Fig. 4b). The results were further validated by RT-PCR (Fig. 3c) and immunoblot analysis (Fig. 3d). Taken together, these results validate HMGB2 as a positive regulator of glycolysis.

### HMGB2 is positively associated with cancer cell growth and the Warburg effect in vivo

**Fig. 5:**
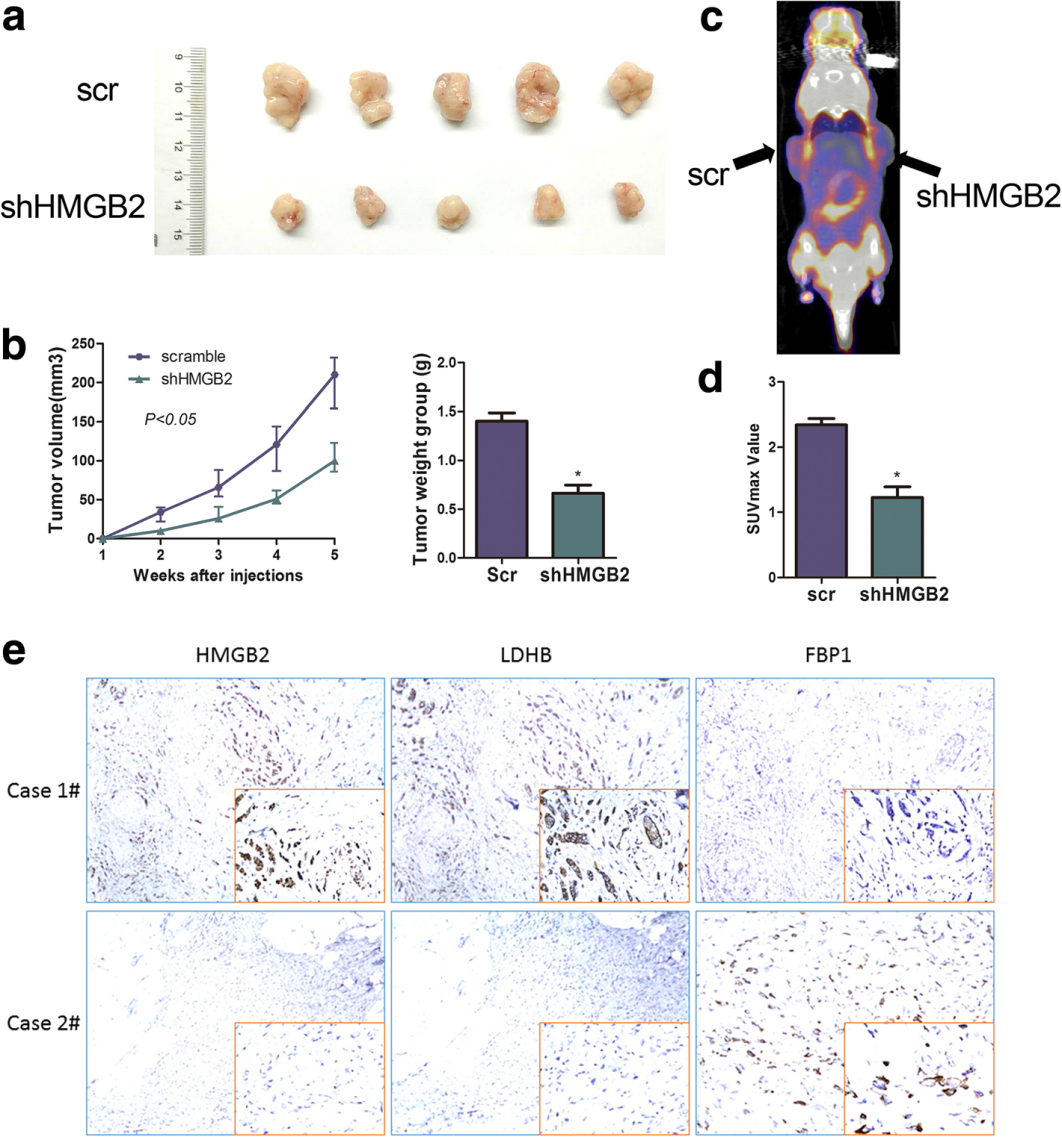
HMGB2 is positively associated with cancer cell growth and the Warburg effect in vivo. MCF-7-scramble/MCF-7-shHMGB2 breast cancer cells were injected subcutaneously into the right or left forelimb of five nude mice (5 × 10^6^ cells per sample for each mouse). Gross tumors in the mice (**a**), and tumor growth curves and weights (**b**) are shown. **c** Representative photographs of 18F–FDG PET/CT scans of MCF-7-scramble/MCF-7-shHMGB2 mice. **d** The SUVmax was lower in the LoVo-shFOXC1 group than in the control group (*P < 0.05). **e** Immunohistochemistry showed that HMGB2 expression is negatively correlated with FBP1 expression, but positively correlated with LDHB expression in patients’ samples

To confirm the oncogene role of HMGB2 in cancer cell growth and the Warburg effect in vivo, we performed tumorigenesis assays in nude mice by subcutaneous injection of MCF-7-scramble/MCF-7-shHMGB2 breast cancer cells. Tumors derived from MCF-7-shHMGB2 cells were significantly smaller and lighter than tumors derived from control cells (*P < 0.05*) (Fig. 5a and b).

High uptake of 18F–FDG by tumors has been suggested as an indicator of the Warburg effect and the PET/CT imaging system was developed as a powerful diagnostic tool based on this glycolysis hypothesis. All mice underwent evaluation by a 18F–FDG PET/CT scan before being sacrificed. The results showed that the SUVmax was lower in MCF-7-knockdown mice than in their paired control group (Fig. 5c and d).

To further confirm the effect of HMGB2 on the expression of the glycolytic enzymes LDHB and FBP1, IHC staining was performed to examine the correlation between HMGB2 with LDHB and FBP1 in breast cancer tissues. Our results demonstrated that HMGB2 expression is positively correlated with expression of LDHB and negatively correlated with FBP1 expression (Fig. 5e).

### HMGB2 transcription regulation of LDHB and FBP1 activity

**Fig. 6:**
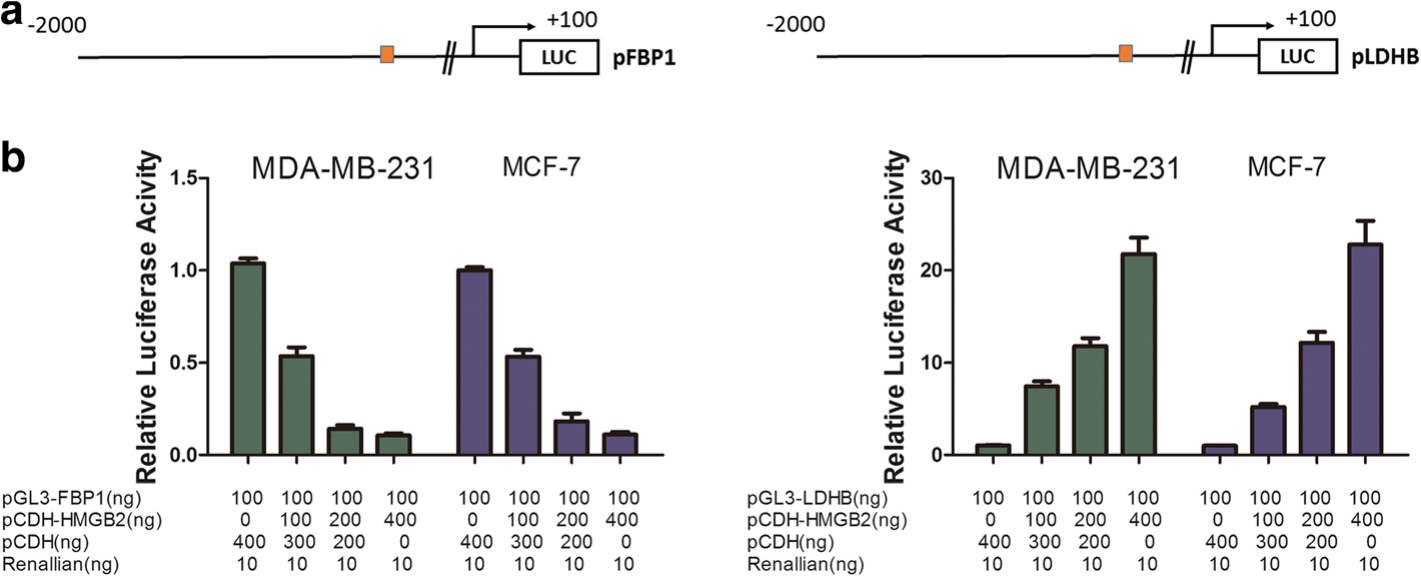
HMGB2 transcriptionally regulates LDHB and FBP1 activities. **a** Demonstrates the schematic graph of the LDHB and FBP1 promoter. **b** HMGB2 can regulate transcription activities of the promoter of LDHB and FBP1

Because HMGB2 expression correlates with expression of LDHB and FBP1 at both transcriptional and protein levels, we hypothesized that HMGB2 could bind to the promoters of LDHB and FBP1 and regulate gene expression. To test this, we obtained luciferase reporter plasmid (pGL3–E-cadherin–Luc) containing a segment of the human LDHB or FBP1 promoter, respectively (Fig. 6a). We observed that when pCDH-HMGB2 was cotransfected into MDA-MB-231 and MCF-7 cells, luciferase activities of LDHB increased at dose dependent, while the activities of FBP1 decreased with dose dependent compared with cells cotransfected with reporter or control (pCDH) vectors (Fig. 6b). These results suggest that HMGB2 is involved in the transcriptional regulation of LDHB and FBP1 expression.

## Discussion

Studies of prognosis and of prognostic factors to predict the risk of recurrence and metastasis for breast cancer patients are intriguing and could affect clinical practice. Established biomarkers such as ER, PR, and HER-2 have already been proven to play significant roles in prognosis and selection of personalized therapy for patients. As such, many previous studies have sought to improve the management of patient care and to understand the biology of breast cancer. In the present study, we focused on the important oncogene HMGB2 and revealed for the first time the functional role of HMGB2 in regulating cell proliferation and the Warburg effect in breast cancer tissues. We demonstrated that strong HMGB2 expression was associated with more aggressive tumor features and reduced OS and DFS in patients. Furthermore, HMGB2 was a positive regulator of proliferation and glycolysis in breast cancer cells. Hence, our study provides significant insights into the novel roles of HMGB2 as a promoting factor of breast cancer progress through regulating of proliferation and glycolysis.The Warburg effect, also known as aerobic glycolysis, is a shift from oxidative phosphorylation to glycolysis, and is considered to be a hallmark of cancer development and progression [26, 27]. It confers a growth advantage on cancer cells by providing anabolic precursors and raw molecules for de novo synthesis and also by minimizing the production of reactive oxygen species in the mitochondria [28]. The Warburg effect is associated with the up- or down-regulated expression of a series of rate-limiting enzymes [27]. Recent studies proved that some oncogenes and tumour suppressors, such as PTEN, HIF-1a, Myc, KLF4, FOXM1, and Gas1 regulate glycolysis in cancer cells [29–31]. Using RT-PCR screening, we found that HMGB2 could promote proliferation and glycolysis by transcriptional regulation of the glycolytic enzymes LDHB and FBP1. HMGB2 is not a classic transcription factor, but it can bend and loop DNA, enhancing flexibility and promoting activities on various gene promoters by enhancing transcription factor binding and/or bringing distant regulatory sequences into close proximity [32–34]. Specifically, we used RT-PCR analysis to initial the mechanism study, there might potential post-translational modification that mediated this phenotype.

## Conclusions

We have defined in this study a novel function of HMGB2 in breast cancer. HMGB2 expression plays important roles in breast cancer progression by regulating proliferation and the Warburg effect by transcriptional regulation of LDHB and FBP1 activity. The frequent upregulation of HMGB2 expression in human breast cancer cells highlights its potential as a novel therapeutic target for this cancer.

## Abbreviations

DFS: Disease free survival
FBP1: Fructose-Bisphosphatase 1.
HMGB2: High-mobility group box 2
LDHB: Lactate Dehydrogenase B
OS: Overall Survival
